# The Evolving Contribution of Cannabis Exposure to Peanut Allergy Development: A Case Report

**DOI:** 10.7759/cureus.106229

**Published:** 2026-03-31

**Authors:** Danielle Ben-Shoshan, Nha Uyen Nguyen Luu

**Affiliations:** 1 Health Sciences, Laurentian University, Sudbury, CAN; 2 Medicine, Université de Montréal, Montreal, CAN

**Keywords:** allergy, cannabis, immunology, mugwort, peanut allergy

## Abstract

Nonspecific lipid transfer proteins are small, heat-stable proteins found in a variety of plant foods and, although once thought to be largely confined to Mediterranean populations, are increasingly recognized as clinically relevant allergens in diverse geographic regions, including North America. We describe a two-year-old boy who developed rapid-onset erythema after peanut ingestion, followed weeks later by recurrent eyelid swelling after outdoor exposure. Evaluation demonstrated sensitization to peanut with elevated Ara h 9, along with sensitization to mugwort and cannabis in the context of passive cannabis smoke exposure. This case suggests that both mugwort allergy and cannabis sensitivity may have contributed to Ara h 9 sensitization and subsequent peanut allergy. Importantly, the present case also presents novel insight into the unexpected, early onset of lipid transfer protein sensitization in children.

## Introduction

Nonspecific lipid transfer proteins (nsLTPs) are small, highly stable plant-derived proteins that function in plant defense and are recognized as potent allergens [1]. These panallergens are found across various plant-based foods, including fruits, vegetables, nuts, seeds, and legumes [2,3].

While LTP-mediated food allergies have been well documented in Southern Europe, particularly in association with primary sensitization to Pru p 3 from peach, these allergies are now being recognized more widely, including in North America, likely due in part to shifting environmental factors such as climate change, which may increase allergen exposure and alter sensitization patterns due to the spread of southern climate-related allergens to the Northern Hemisphere [2].

Recent evidence has broadened the understanding of LTP sensitization pathways, implicating non-food sources such as mugwort pollen (Art v 3) and marijuana (Can s 3) as potential primary sensitizers, including via passive inhalation (i.e., pollen, second-hand smoke inhalation) [4].

Ara h 9, a peanut-derived LTP, is also emerging as a significant allergen in peanut-allergic individuals, particularly in those sensitized via the LTP pathway rather than the traditional peanut allergens of Ara h 1, 2, and 3 [5].

We present a case highlighting a rare LTP syndrome presentation in a North American pediatric patient with multi-food allergic reactions and suspected airborne sensitization through second-hand cannabis smoke inhalation. This case underscores the need for heightened clinical awareness of nsLTP-mediated food allergy beyond Southern-hemispheric populations and offers insight into diagnostic and management strategies in a current landscape where climate change is altering the distribution and timing of allergens, creating new challenges for patients and clinicians alike [6]. Furthermore, this case may point to the importance of continued evaluation of public health policies and legislation related to cannabis use and pediatric exposure, particularly in light of potential allergenic risks identified in newly emerging research.

This article was previously presented as an oral presentation at the 2024 Canadian Society of Allergy and Clinical Immunology.

## Case presentation

A two-year-old boy presented to our hospital in August 2023 after having a sudden allergic reaction minutes after ingestion of a snack product that was later identified as containing peanut-derived ingredients. The reaction included redness and discomfort in the perioral region, but he showed no signs of respiratory distress, gastrointestinal problems, or anaphylaxis. He had no known history of adverse peanut reaction.

About three weeks later (September 2023), the patient experienced several episodes of bilateral eyelid swelling that occurred shortly after playing outside. During one of these episodes, his mother administered a 0.15 mg dose of intramuscular epinephrine using an autoinjector due to concern for a possible allergic reaction. The consistent timing of these episodes following outdoor exposure raised suspicion for an environmental trigger, with aeroallergens such as mugwort considered a likely contributing factor.

**Figure 1:**
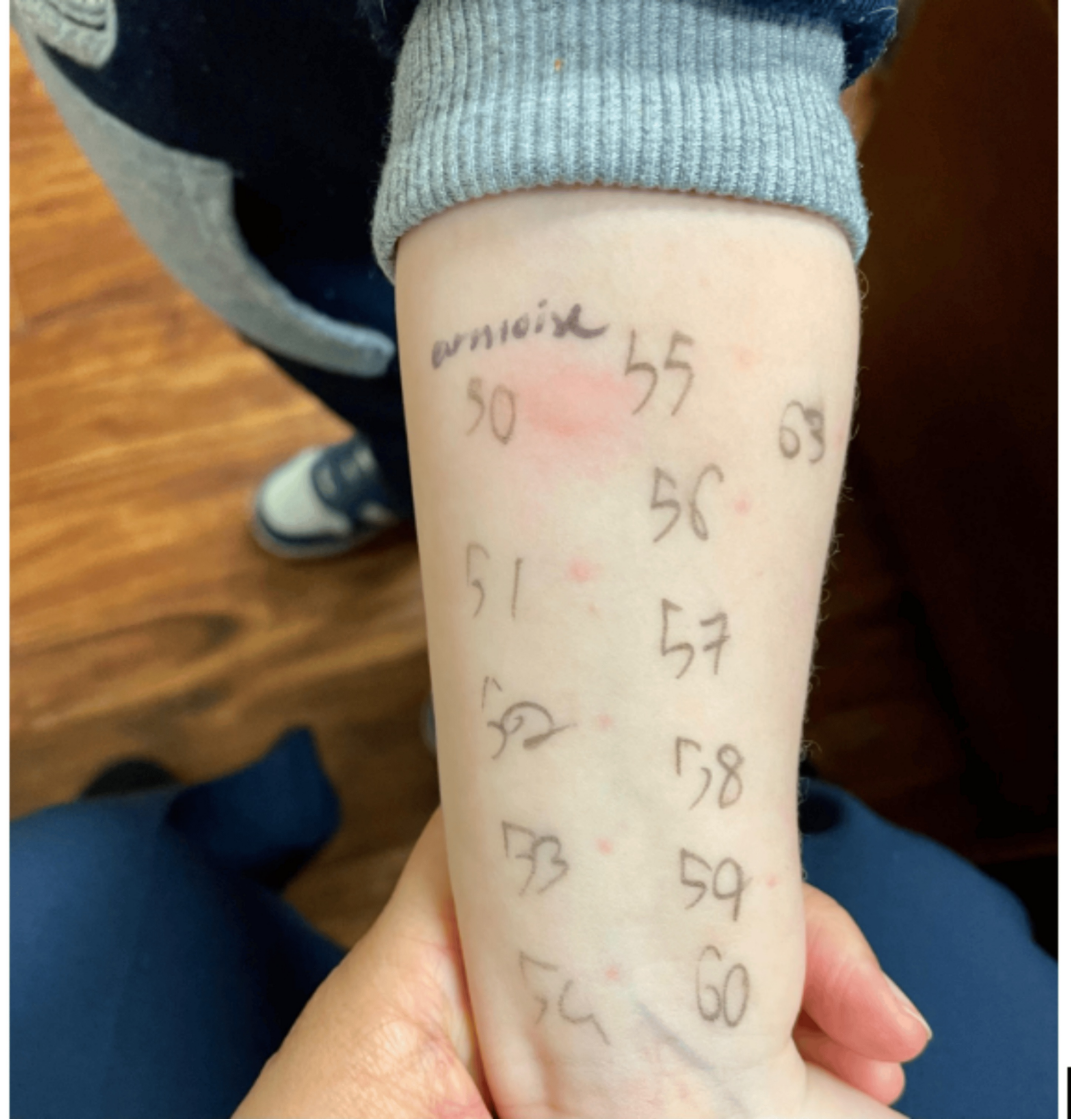
Patient skin test results for peanut, mugwort, and cannabis.

The patient was subsequently initiated on a peanut oral immunotherapy (OIT) protocol. However, the treatment was discontinued due to the development of recurrent vomiting and abdominal pain following OIT dosing. No endoscopy was performed at that time. A detailed social and environmental history revealed that the child was frequently exposed to passive cannabis smoke in the household, specifically from the maternal grandmother, who used cannabis in the home setting. There was no reported direct contact or ingestion of cannabis. Skin prick testing (SPT) was performed, which yielded positive reactions to peanut, mugwort, and cannabis extracts (Figure 1).

Laboratory investigations are summarized in Table 1. Evaluation demonstrated peripheral eosinophilia with a normal serum tryptase level and total serum IgE within the reference range. Allergen-specific IgE testing showed sensitization to peanut, with a predominance of Ara h 9, while Ara h 3 and Ara h 8 were not detected. This pattern supports lipid transfer protein-mediated sensitization.

**Table 1:** Summary of laboratory investigations demonstrating Ara h 9-mediated sensitization.

Laboratory parameter	Result (qualitative)	Value	Reference range
Peripheral eosinophil count	Increased	600 cells/µL	0-500 cells/µL
Serum tryptase	Normal	5.7 ng/mL	<11.4 ng/mL
Total serum IgE	Normal	30 kU/L	≤60 kU/L (age-adjusted)
Peanut-specific IgE	Increased	5.20 kU/L	<0.35 kU/L
Ara h 9-specific IgE	Increased	7.98 kU/L	<0.35 kU/L
Ara h 3-specific IgE	Not detected	<0.10 kU/L	<0.35 kU/L
Ara h 8-specific IgE	Not detected	<0.10 kU/L	<0.35 kU/L

At the time of this writing, the clinician advised the child’s guardian to avoid peanuts and relevant cross-reactive foods. The clinician discussed environmental controls with the family, particularly concerning potential passive cannabis smoke inhalation. The case remains under follow-up with pediatric allergy and immunology for further evaluation and management planning.

## Discussion

This case provides a unique perspective on the sensitization pattern observed in a pediatric patient with documented exposure to cannabis. The detection of Ara h 9 sensitization is particularly noteworthy, as this lipid transfer protein (LTP) allergen is more frequently associated with peanut allergy in Mediterranean and Southern European populations, where cross-reactivity with plant-derived LTPs (such as peaches, mugwort) is well documented [7]. In contrast, in North America, Ara h 9 sensitization has historically been less common, with LTP-containing sources such as mugwort pollen and peaches being less common due to northern climatic growth patterns [8]. However, with ongoing climate change, particularly rising temperatures, altered precipitation patterns, and extended growing seasons, there is concern that the geographic range, pollen load, and allergenic potency of LTP-containing plants will expand, potentially increasing Ara h 9 sensitization rates in North American populations over time [9].

In addition, it was notable that this was an early pediatric case (two years of age) of Ara h 9 sensitivity, which is unusual considering that LTP-containing plant sensitization typically develops after four years of age due to cumulative seasonal exposure [10]. As such, we hypothesize that mugwort and cannabis may share nonspecific LTP allergens, such that environmental exposure to cannabis could act as an early sensitizing agent for both peanut (via Ara h 9) and mugwort. In this context, sustained cannabis exposure, particularly in a household setting, may promote earlier immune recognition and sensitization to these allergens. This mechanism could account for the unusual presentation of Ara h 9 sensitization at a pediatric age, in parallel with early-onset mugwort allergy.

It is important to note that in this case study, we are not confirming clinical cannabis allergy, but rather suggesting possible sensitization. The patient did not exhibit immediate hypersensitivity symptoms (e.g., rhinorrhea, conjunctival symptoms, wheeze) upon exposure to cannabis smoke. However, the absence of clinical reactivity does not exclude immunologic sensitization, which reflects detectable allergen-specific IgE without necessarily manifesting reproducible clinical symptoms upon exposure [11].

From a broader public health perspective, these findings intersect with an evolving social and legislative matter concerning the legalization of cannabis over the past decade. Since the 2018 legalization of cannabis in Canada, opportunities for both intentional and unintentional exposure to cannabis smoke have expanded [12]. This is particularly concerning among children living in multi-unit housing or households where cannabis is smoked by a family member or in a neighboring unit [13]. Given the rapid commercialization of cannabis products, the potential for widespread passive exposure is likely to increase unless public health policy and practice preventive measures are considered [14].

Considering the findings of this case and the limited but growing research on cannabis exposure and pediatric allergy, there is a compelling need for proactive public health discussion [15,16]. Future public health policy should explicitly address the potential implications of cannabis legalization and commercialization on child health. This includes developing strategies to limit passive exposure in residential settings, childcare environments, and public spaces. Policymakers and researchers should work collaboratively to ensure that emerging evidence is effectively translated into regulatory frameworks and that such frameworks remain adaptable as the science evolves. Clear communication between research, policy, and practice is essential to protect vulnerable populations, promote health equity, and inform evidence-based regulations aimed at minimizing preventable exposures.

## Conclusions

This case describes a rare instance of Ara h 9 sensitization in a very young child, occurring alongside prolonged cannabis exposure within the home. The sensitization profile, combined with known cross-reactivity among plant-derived lipid transfer proteins, raises the possibility that cannabis may act as a source of nonspecific LTP allergens, associated with an earlier onset of sensitization to both peanut and mugwort. Ara h 9 allergy is most often reported in Mediterranean regions and tends to emerge later in childhood or adulthood; the timing and context here suggest an alternate pathway of sensitization. Such pathways may become increasingly relevant in areas where climate and environmental shifts influence pollen distribution, load, and allergenicity. In clinical allergy practice, taking a thorough environmental history, including potential cannabis exposure, may help identify unusual sensitization patterns in pediatric patients. Additional studies are required to explore the biological mechanisms underlying these associations as well as to assess their broader public health implications. The observations from this case also support the development of evidence-based regulations to reduce passive cannabis exposure, particularly in environments where children may be routinely present.
